# The Transcription-Repair Coupling Factor Mfd Prevents and Promotes Mutagenesis in a Context-Dependent Manner

**DOI:** 10.3389/fmolb.2021.668290

**Published:** 2021-05-20

**Authors:** Laura A. Lindsey-Boltz, Aziz Sancar

**Affiliations:** Department of Biochemistry and Biophysics, University of North Carolina School of Medicine, Chapel Hill, NC, United States

**Keywords:** mutation frequency decline (MFD), nucleotide excision repair (NER), excision repair-sequencing (XR-seq), transcription-coupled repair (TCR), uvrABC excinuclease, UvrD

## Abstract

The *mfd* (mutation frequency decline) gene was identified by screening an auxotrophic *Escherichia coli* strain exposed to UV and held in a minimal medium before plating onto rich or minimal agar plates. It was found that, under these conditions, holding cells in minimal (nongrowth) conditions resulted in mutations that enabled cells to grow on minimal media. Using this observation as a starting point, a mutant was isolated that failed to mutate to auxotrophy under the prescribed conditions, and the gene responsible for this phenomenon (mutation frequency decline) was named *mfd*. Later work revealed that *mfd* encoded a translocase that recognizes a stalled RNA polymerase (RNAP) at damage sites and binds to the stalled RNAP, recruits the nucleotide excision repair damage recognition complex UvrA_2_UvrB to the site, and facilitates damage recognition and repair while dissociating the stalled RNAP from the DNA along with the truncated RNA. Recent single-molecule and genome-wide repair studies have revealed time-resolved features and structural aspects of this transcription-coupled repair (TCR) phenomenon. Interestingly, recent work has shown that in certain bacterial species, *mfd* also plays roles in recombination, bacterial virulence, and the development of drug resistance.

## Mutation Frequency Decline

The “mutation frequency decline” (MFD) phenomenon was discovered by Evelyn Witkin 65 years ago (Witkin, 1956). Notably, this was 4 years before the discovery of *Escherichia coli* RNA polymerase (RNAP; Hurwitz et al., 1960), and several years before it was even known that thymine dimers were the major UV lesions in *E. coli* DNA (Wacker et al., 1962) and that such dimers are repaired in *E. coli* either by a visible light–dependent photoreactivating enzyme (Rupert et al., 1958), later named photolyase (Sancar, 2008), or by another mechanism called nucleotide excision repair (NER; Setlow and Carrier, 1964). The MFD phenomenon describes the observation that the yield of UV-induced mutations in specific auxotrophic *E. coli* strains is dependent on the number of nutrients present during the first cell division after irradiation. In other words, when Witkin briefly held the UV-irradiated auxotrophic strain for a few minutes under a condition where protein synthesis was inhibited before plating them onto rich agar plates, a decrease in the frequency of mutations was observed. She found that protein synthesis-inhibiting posttreatments that caused MFD, such as incubation in low nutritional media or the addition of the protein synthesis inhibitor chloramphenicol, did not affect the overall survival or change the yields of other kinds of mutations. Witkin went on to isolate a mutant *E. coli* strain, *mfd*-, that failed to mutate to auxotrophy under the prescribed conditions (Witkin, 1966), and then, 25 years later, she sent this strain to the Sancar Lab where Christopher Selby determined that the strain lacks the transcription-repair coupling activity (Selby et al., 1991) that he had been characterizing (Selby and Sancar, 1990, 1991). Thus, Mfd was the long sought-after *E. coli* transcription-repair coupling factor (TRCF) (reviewed in Selby, 2017).

## Transcription-Coupled Repair

Nucleotide excision repair is a versatile DNA repair pathway that removes all types of DNA-helix-distorting lesions (Sancar et al., 2004). NER occurs *via* two pathways: the predominant pathway, global genome repair (GGR), functions throughout the whole genome and a sub-pathway, transcription-coupled repair (TCR), specifically repairs the template strand of genes by acting upon lesions that block transcription by RNAP. NER plays a critical role in genome integrity, and both NER pathways can be found in all domains of life; however, the excision repair proteins in prokaryotes are not evolutionarily related to those in eukaryotes.

**FIGURE 1:**
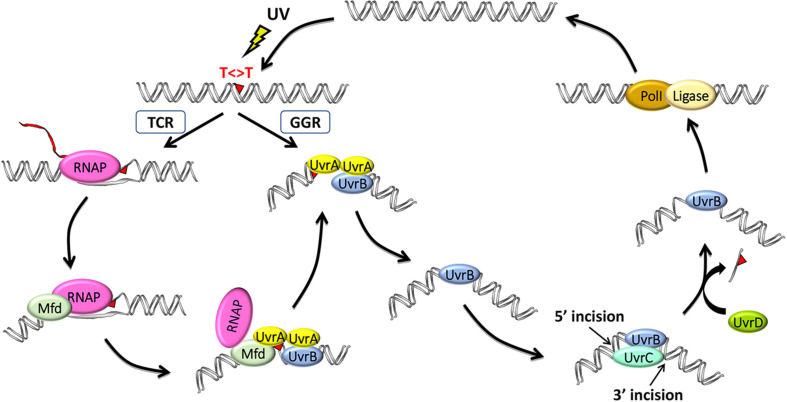
Model for the two nucleotide excision repair pathways in *E. coli*: general global repair (GGR) and transcription-coupled repair (TCR). UV light induces thymine dimers in DNA which are either directly recognized by UvrA_2_B in the GGR pathway or indirectly recognized by RNA polymerase (RNAP) in the TCR pathway. Elongating RNAP stalls when it encounters a dimer in the template strand and recruits the mutation frequency decline (Mfd) translocase, which, in turn, removes RNAP while recruiting UvrA_2_B. The two pathways then converge after these initial damage-recognition steps, and UvrA_2_ dissociates, leaving a stable preincision complex consisting of UvrB bound to damaged DNA, which now has an altered structure. UvrC is recruited to generate the coupled dual incisions, and UvrD removes UvrC and the damaged oligonucleotide. Repair is completed by synthesis and ligation of the repair patch by DNA polymerase I (PolI) and DNA ligase, respectively.

Using defined *in vitro* systems, the Sancar Lab has elucidated the reaction mechanisms of both global and TCRs in *E. coli* (reviewed in Sancar, 2016). Except for differences in initial damage recognition, both NER pathways are essentially the same in that they utilize the excision repair proteins UvrA, UvrB, and UvrC to perform dual incisions that remove the DNA damage in the form of 12- to 13-nucleotide oligomers and then utilize the proteins UvrD (helicase II), DNA polymerase I, and DNA ligase to release the excised oligomer, resynthesize the resulting gap, and ligate, respectively (Figure 1).

Global NER is initiated when the damage recognition factor, UvrA, which exists as a dimer together in a complex with UvrB (denoted UvrA_2_UvrB), facilitates the formation of a stable UvrB–DNA complex in an ATP hydrolysis–dependent reaction (Hu et al., 2017). UvrA then disassociates from the complex, and UvrB recruits the UvrC endonuclease to the damage site. UvrC is a multidomain nuclease, which first incises the DNA at the 3rd or 4th phosphodiester bond 3′ to the lesion *via* its GIY-YIG catalytic domain and then at the 7th phosphodiester bond 5′ to the lesion using its C-terminal RNase H-like catalytic domain. The UvrD helicase then displaces the excised damaged strand.

Damage recognition is the rate-limiting step in NER, and some damage, such as the UV light–induced cyclobutane pyrimidine dimer (CPD), causes only minimal distortion to the DNA double helix and is thus poorly recognized by GGR (Hu et al., 2017). As a result, repair of such damage is greatly facilitated by RNAP scanning the DNA to initiate the repair of these lesions. When RNAP encounters DNA damage, it forms a stable complex at the damage site that inhibits repair by interfering with the access of UvrA_2_UvrB to the damage (Selby and Sancar, 1990). Mfd recognizes stalled RNAP and displaces it from the damage site while concomitantly recruiting UvrA_2_UvrB (Selby and Sancar, 1993a). Even though the Mfd protein has been extensively studied for nearly three decades since it was cloned (Selby and Sancar, 1993a) and characterized (Selby and Sancar, 1993b, 1994, 1995a,b) by Selby, several recent reports have significantly advanced our understanding of Mfd, including discoveries from whole-genome analyses (Adebali et al.,2017a,b; Ragheb et al., 2021), as well as from structural (Brugger et al., 2020; Kang et al., 2021) and single-molecule (Fan et al., 2016; Ho et al., 2018, 2020; Ghodke et al., 2020) studies which will be reviewed here.

## Recent Advances: Whole-Genome Studies

Transcription-coupled repair in *E. coli* was first described by the Hanawalt Lab when they reported 10-fold faster repair of the transcribed strand of the *lac* operon (Mellon and Hanawalt, 1989). Although this was subsequently confirmed with the analysis of several other *E. coli* genes in the 30 years since the original report, a significant advance in the field occurred when the Sancar Lab recently developed a method named eXcision Repair-sequencing (XR-seq) to map NER events throughout the whole genome at single nucleotide resolution (Hu et al., 2015) and employed this method to map CPD repair in *E. coli* (Adebali et al.,2017a,b). Briefly, the XR-seq method consists of purifying excised damaged oligos by immunoprecipitation with CPD-specific antibodies, ligating the isolated DNA to adapters, repairing the CPDs by photoreactivation, amplifying the DNA by PCR, and then next-generation sequencing and mapping the reads to the genome (Hu et al., 2017).

The Sancar Lab generated XR-seq CPD repair maps from several different *E. coli* strains, including *mfd*- and *uvrD*-, to assess the roles of these proteins in TCR (Adebali et al.,2017a,b). The maps revealed a rather complex genome-wide pattern of repair in the regions of annotated genes because of the widespread antisense transcription throughout most of the *E. coli* genome. Nevertheless, it was clear that Mfd is required for TCR. In fact, they found that the nontemplate strand is preferentially repaired in the *mfd*- strain, likely due to the interference of damage recognition by UvrA_2_UvrB when RNAP is stalled at the damage sites in the template strand. In contrast, TCR slightly increased in the absence of UvrD, consistent with the role of UvrD in the catalytic turnover of the Uvr(A)BC excision nuclease. Following the dual incisions, the UvrB-UvrC-excised oligomer complex remains bound to the duplex, and this complex is displaced by the UvrD helicase to release UvrC, the limiting repair factor, for new rounds of repair (Figure 1). In the *uvrD-*strain, there is a higher yield of recovered excised oligos due to their protection from nucleases when complexed with UvrB–UvrC, yet there is lower overall repair, and the slight increase in TCR seen in the *uvrD-* strain is due to Mfd facilitating the first and the only round of repair in the template strand. These same findings were also observed when XR-seq was used to analyze the *lac* operon under conditions where *lacZ* is either not expressed, in glucose-containing medium, or expressed, by the addition of isopropyl β-d-1-thiogalactopyranoside (IPTG; Adebali et al., 2017b). They found that the transcribed strand of *lacZ* is repaired ∼5-fold faster in the wild-type strain. In the *mfd-* strain, TCR was abolished and the nontranscribed strand was repaired in a higher level, whereas in the *uvrD-* strain, TCR was slightly enhanced although the overall repair was reduced.

As previously mentioned, NER is evolutionarily conserved, and indeed, the generation of XR-seq repair maps of parental and *uvrD*- strains of another prokaryote, *Mycobacterium smegmatis*, which is a very close relative of the human pathogen *Mycobacterium tuberculosis*, demonstrated that the TCR repair mechanism in Mycobacteria is the same as in *E. coli* (Selby et al., 2020). In conclusion, genome-wide studies provide detailed repair maps that complement the curated transcription maps of *E. coli* and *M. smegmatis* and confirm a central role of the Mfd protein in coupling transcription to repair in prokaryotes.

A very recent *E. coli* whole-genome analysis from Houra Merrikh’s lab mapped Mfd-associated genomic loci using chromatin immunoprecipitation followed by high-throughput sequencing (ChIP-seq) and found a very high correlation (*r* = 0.98) between sites bound by Mfd and those bound by RNAP (Ragheb et al., 2021). Interestingly, this study was performed in the absence of exogenous DNA damage, and the Mfd-bound sites correlated (*r* = 0.6) with the sites of the *E. coli* RNA secondary structure as determined by parallel analysis of RNA structure (PARS-seq). They compared RNAP chromatin association in the presence and absence of Mfd and found that 40% of the genes that had at least a twofold increase in RNAP association in the *mfd-*strain contained a regulatory RNA or structural element. This, together with their other results from experiments in *Bacillus subtilis*, led them to conclude that Mfd regulates RNAP in hard-to-transcribe regions such as those with structured RNAs. These and other discoveries have direct implications on the role of Mfd in bacterial virulence and the development of drug resistance and will be discussed further below.

## Recent Advances: Mfd Structural Studies

**FIGURE 2:**
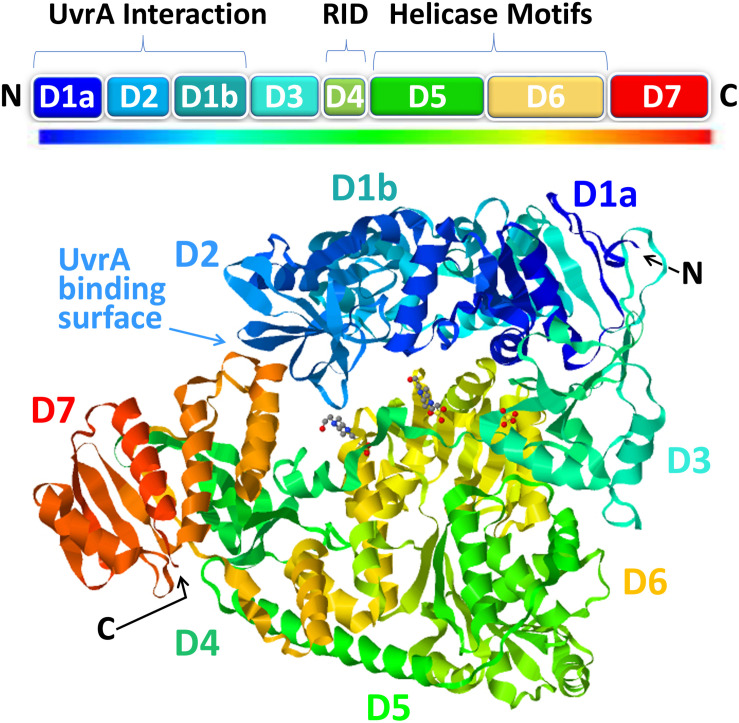
The structure of mutation frequency decline (Mfd). The modular Mfd protein consists of eight domains indicated as boxes in the cartoon (top) and as a rainbow ribbon representation of the crystal structure [PDB ID: 2EYQ (Deaconescu et al., 2006)] viewed with JSmol (bottom). The N-terminus (N) contains the UvrA interaction region (D1a, D2, D1b) which is structurally homologous to the region of UvrB that binds to UvrA. This region of the protein is sequestered in a locked state *via* interactions with the D7 autoinhibitory domain in the C-terminus (C). Upon interaction with stalled RNAP, the D4 domain, containing the RNA polymerase (RNAP)-interacting domain (RID), binds to the β’ subunit of RNAP which triggers ATP hydrolysis by the helicase motifs in D5-D6 and subsequent DNA translocation and rearrangements ultimately resulting in release of the nascent RNA, removal of RNAP, and recruitment of UvrA_2_B repair factors.

Recent progress on the structural biology of Mfd has provided significant details and resolution to our understanding of how this enzyme functions in TCR. Mfd is a multidomain protein composed of eight domains, namely, D1a, D1b, and D2–D7 (Figure 2). The x-ray crystallography structure of full-length Mfd (Deaconescu et al., 2006) showed that it normally exists in a repressed conformation with its C-terminal D7 domain interacting with its N-terminal D2 domain, which is homologous to the UvrA-interacting domain of UvrB. Multiple large conformational changes occur in Mfd when it engages with and displaces RNAP and while recruiting UvrA_2_UvrB to the DNA damage, and recent cryo-electron microscopy (cryo-EM) studies have provided enough high-resolution images of the intermediates to provide a clear understanding of this cycle (Brugger et al., 2020; Kang et al., 2021). The cryo-EM structures beautifully illustrate how Mfd is remodeled from its repressed conformation to expose the UvrA-interacting surface in D2, which is hidden during most of the remodeling process to prevent premature interactions with UvrA_2_UvrB. Domain D4 of Mfd, which contains the RNAP interaction domain (RID), interacts with the β-subunit of RNAP, and the EM images demonstrate how Mfd engages with the RNAP bound to DNA damage (Kang et al., 2021). Although initial binding of the RID to the RNAP does not require conformational changes, at least one round of ATP hydrolysis is required for Mfd to form a stable complex with RNAP, and this allows tethering of the Mfd translocation module (domains D5 and D6) to the upstream duplex DNA (Kang et al., 2021). Domains D5 and D6 of the Mfd are homologous to the RecG bacterial motor protein that couples ATP hydrolysis to double-stranded DNA translocation; however, unlike RecG and other helicases, Mfd cannot separate the DNA strands (Selby and Sancar, 1995b). After the initial interaction of Mfd with stalled RNAP, a series of stepwise dynamic conformational changes is triggered resulting in Mfd completely encircling the upstream duplex DNA and culminating in the ATP-hydrolysis-powered disruption of the RNAP (Kang et al., 2021).

## Recent Advances: Mfd Single-Molecule Studies

Single-molecule approaches can be very useful in studying multicomponent, multistep reactions such as TCR. *In vitro* single-molecule experiments allow one to answer questions such as which proteins are present, what are their stoichiometries, and how quickly do they come and go (Strick and Portman, 2019). Many observations from *in vitro* Mfd single-molecule studies have confirmed and added a more detailed understanding to aspects of the mechanism that was determined by Selby’s original population-averaging *in vitro* biochemistry experiments (Selby and Sancar,1993a,b, 1994, 1995a,1995b), such as the observation that Mfd binds stalled RNAP and uses the energy from ATP hydrolysis to displace the stalled RNAP from DNA (Howan et al., 2012) and that the displacement of RNAP from DNA is accompanied by the loss of the nascent RNA (Graves et al., 2015; Kang et al., 2021). One surprising result is that the displaced RNAP remains in a long-lived complex with Mfd on the DNA, and in the absence of DNA damage, this Mfd–RNAP complex is capable of translocating thousands of base pairs in the same direction as the initial transcription (Howan et al., 2012; Graves et al., 2015). The interaction of Mfd with DNA induced bending or wrapping of the DNA, and it has been proposed that the high processivity of Mfd translocation is due to this topological wrapping. In a different single-molecule study, Mfd was added to DNA alone and was found to translocate for a few hundred base pairs (Le et al., 2018). However, as previously discussed, Mfd is thought to exist in a repressed state when not bound to stalled RNAP (Deaconescu et al., 2006, 2012) and has been shown to have only weak DNA-binding activity on its own (Selby and Sancar, 1995a), and thus, the physiological relevance is unclear. Single-molecule studies also showed that Mfd can rescue RNAP at pause sites, but more severe obstacles to RNAP movement such as DNA damage lead to eventual transcription termination (Le et al., 2018). The addition of either UvrA_2_ or UvrA_2_B to the single-molecule system arrested the translocating Mfd–RNAP complex, and then, both Mfd and RNAP were released from the DNA (Fan et al., 2016). Then, with the further addition of UvrC, incision was observed in the damaged DNA, and the kinetics was in agreement with previous estimates of ∼3-fold faster repair by TCR than by GGR (Fan et al., 2016).

*In vivo* single-molecule experiments are very useful for analyzing the diffusion of proteins inside cells as they search for and bind to their targets (Strick and Portman, 2019). The van Oijen Lab fluorescently labeled Mfd in live *E. coli* and found that it interacts with RNAP even in the absence of exogenous DNA damage (Ho et al., 2018). The authors proposed that the interactions involved naturally stalled RNAP because they were enriched in the presence of a drug that stalls RNAP, they were absent in cells treated with a transcription inhibitor, and the presence of UvrA shortened the lifetime of the Mfd-RNAP-DNA complexes. In back-to-back follow-up reports, they analyzed fluorescently labeled Mfd in UV-irradiated cells and also analyzed fluorescently labeled UvrA (Ghodke et al., 2020; Ho et al., 2020). They reported that the lifetime of the Mfd-RNAP-DNA complex decreased from ∼18 s, in the absence of exogenous DNA damage, to 12 s in UV-irradiated cells and that this mirrored what was seen with fluorescently labeled UvrA, and the lifetime of UvrA was dependent on the presence of Mfd indicating that the proteins function together (Ghodke et al., 2020). In the companion report, they employed ATPase mutants of UvrA and damage-recognition mutants of UvrB to analyze UvrA_2_B recruitment and Mfd dissociation *in vivo*. As predicted from earlier genetic and biochemistry studies, they found that Mfd is stably arrested on DNA in both mutant backgrounds relative to wild-type cells and concluded that Mfd dissociation is coupled with successful loading of UvrB (Ho et al., 2020). In conclusion, the *in vitro* and *in vivo* single-molecule studies on *E. coli* TCR provide detailed resolution that advances our understanding of this complex, multicomponent, and multistep reaction.

## Roles of Mfd in Recombination, Bacterial Virulence, and Development of Drug Resistance

As discussed above, the role of Mfd in *E. coli* TCR has been extensively characterized; however, many of the other reported cellular functions of Mfd are less well understood (Strick and Portman, 2019). There are reports suggesting that Mfd can enhance prokaryotic virulence and survival *via* the promotion of mutations in various genes involved in cell wall biosynthesis, translation, and transcription and has led to Mfd being called a “proevolutionary factor” (Strick and Portman, 2019) or “evolvability factor” (Ragheb et al., 2019; Brugger et al., 2020); however, there is no clear consensus as to the underlying mechanism. This has become a topic of wide interest due to the clinical implications of possibly targeting Mfd for antimicrobial drug resistance prevention, and recent results that shed some light on the subject will be discussed below.

When Witkin isolated the *mfd-*mutant strain, she reported that it produced ∼5-fold more UV-induced mutations than the parent strain even though it was no more sensitive to UV irradiation than its parent (Witkin, 1966). Witkin’s assay specifically selected mutations in tRNA suppressor genes (Witkin, 1994), but later studies of the *lacI* gene also showed that the UV-induced mutation frequency was reduced ∼5-fold by Mfd and, at one particular site, by more than 300-fold (Oller et al., 1992). Thus, for DNA damage–induced mutagenesis, Mfd clearly functions as an antimutator; however for “spontaneous mutagenesis,” it appears to function as a mutator. For example, the antimicrobial drugs to which resistance develops in an Mfd-dependent manner are not known as DNA-damaging agents, and thus, the function of Mfd was not clear in this phenomenon. Mfd plays a role in recombination (Ayora et al., 1996) and facilitates the generation of R-loops (Portman et al., 2021) which initiate DNA breakage and genome instability (Wimberly et al., 2013), consistent with the notion that Mfd facilitates transcription-associated mutagenesis (Ayora et al., 1996; Jinks-Robertson and Bhagwat, 2014; Gomez-Marroquin et al., 2016; Ragheb et al., 2019; Portman et al., 2021). This has recently gained support from the recent study discussed above (from the Merrikh Lab), showing that Mfd regulates RNAP in hard-to-transcribe regions such as those with structured RNAs (Ragheb et al., 2021). The authors analyzed the Mfd-bound genes and found that they were involved in a variety of cellular functions including toxin–antitoxin systems. Indeed, they went on to show that cell viability is compromised by the overexpression of toxin genes in the absence of Mfd and that the mutation rate of one particular toxin gene is lower by ∼7-fold in *mfd-* cells compared to wild-type cells. In conclusion, it is evident that Mfd plays a role in RNAP transcriptional control at regions of frequent RNAP pausing, and specific regions of the genome may be prone to transcription-associated mutagenesis due to inherent RNA structure. Thus, Mfd prevents and promotes mutagenesis in a context-dependent manner.

## Author Contributions

All authors listed have made a substantial, direct and intellectual contribution to the work, and approved it for publication.

## Conflict of Interest

The authors declare that the research was conducted in the absence of any commercial or financial relationships that could be construed as a potential conflict of interest.

## Abbreviations

Mfd: mutation frequency decline
RNAP: RNA polymerase
NER: nucleotide excision repair
GGR: global genome repair
TCR: transcription-coupled repair
TRCF: transcription-repair coupling factor
CPD: cyclobutane pyrimidine dimer
XR-seq: excision repair-sequencing
ChIP-seq: chromatin immunoprecipitation followed by high-throughput sequencing
RID: RNA polymerase interaction domain
EM: electron microscopy.
